# The Effects of Pulmonary Artery Catheter on the Short-Term Outcomes of Patients Undergoing Off-Pump Coronary Artery Bypass Grafting: A Single-Center Retrospective Study

**DOI:** 10.31083/j.rcm2505183

**Published:** 2024-05-22

**Authors:** Chun-mei Xie, Yun-tai Yao, Wen-hui Qi, Meng-qi Shen, Li-xian He, the Evidence in Cardiovascular Anesthesia (EICA) Group

**Affiliations:** ^1^Department of Anesthesiology, Fuwai Yunnan Hospital, Chinese Academy of Medical Sciences, Affiliated Cardiovascular Hospital of Kunming Medical University, 650000 Kunming, Yunnan, China; ^2^Department of Anesthesiology, Fuwai Hospital, National Center for Cardiovascular Diseases, Peking Union Medical College and Chinese Academy of Medical Sciences, 100037 Beijing, China; ^3^Department of Anesthesiology, Harrison International Peace Hospital, 053000 Hengshui, Hebei, China

**Keywords:** off-pump coronary artery bypass, outcomes, pulmonary artery catheter

## Abstract

**Background:**

Pulmonary artery catheters (PAC) are widely used in patients undergoing off-pump coronary artery bypass (OPCAB) grafting surgery. However, primary data suggested that the benefits of PAC in surgical settings were limited. Therefore, the present study sought to estimate the effects of PAC on the short-term outcomes of patients undergoing OPCAB surgery.

**Methods:**

The characteristics, intraoperative data, and postoperative outcomes of consecutive patients undergoing primary, isolated OPCAB surgery from November 2020 to December 2021 were retrospectively extracted. Patients were divided into two groups (PAC and no-PAC) based on PAC insertion status. Data were analyzed with a 1:1 nearest-neighbor propensity score matched-pair in PAC and no-PAC groups.

**Results:**

Of the 1004 Chinese patients who underwent primary, isolated OPCAB surgery, 506 (50.39%) had PAC. Propensity score matching yielded 397 evenly balanced pairs. Compared with the no-PAC group (only implanted a central venous catheter), PAC utilization was not associated with improved in-hospital mortality in the entire or matched cohort. Still, the matched cohort showed that PAC utilization increased epinephrine usage and hospital costs.

**Conclusions:**

The current study demonstrated no apparent benefit or harm for PAC utilization in OPCAB surgical patients. In addition, PAC utilization was more expensive.

## 1. Introduction

Pulmonary artery catheters (PAC) were introduced in the 1970s by professors Jeremy Swan and William Ganz [1]. Nowadays, the classical PAC has a nearly 50-year history of clinical applications for hemodynamic monitoring. In recent years, PAC have evolved from a device capable of combining static pressure measurements of intermittent cardiac output to a monitoring tool that could continuously measure cardiac output, oxygen supply, demand balance, and right ventricular function [2]. PAC utilization was valuable in guiding treatment in critically ill and complex surgical patients [3, 4, 5, 6], especially for patients undergoing cardiac surgery [7, 8, 9, 10]. Shaw *et al*. [7] reported that PAC utilization was associated with decreased length of stay (LOS), reduced cardiopulmonary morbidity, and increased infectious morbidity but no increase in 30-day in-hospital mortality during adult cardiac surgery. This suggested a potential benefit of PAC utilization in cardiac surgical patients [7].

PAC provided hemodynamic data, which were used to determine treatment measures. The decisive question was whether these treatment measures improve outcomes. For patients undergoing coronary artery bypass graft (CABG) surgery, PAC remains the most commonly used monitoring method among cardiovascular anesthesiologists [8]. For the studies that evaluated the effect of PAC utilization on the outcomes of CABG surgical patients, very few studies reported that the benefits of PAC outweigh the risks [9, 10]; two studies reported a neutral effect of PAC on clinical outcomes [11, 12]; several studies reported the harm of PAC [13, 14, 15, 16, 17]. Given the current use of PAC in off-pump coronary artery bypass (OPCAB) grafting surgery, the present study analyzed records of patients undergoing OPCAB surgery with or without PAC to estimate the impact of PAC use on short-term clinical outcomes.

## 2. Methods

### 2.1 Study Design and Target Population

The Ethical Committee approved the single-center study (2019-1301). Because of the retrospective nature of this study, patient consent was waived. Patients who underwent primary and isolated OPCAB surgery from November 2020 to December 2021 were enrolled in this retrospective cohort study. Exclusion criteria were: (1) patients undergoing OPCAB combined with other valve surgery; (2) endocarditis status; (3) patients with emergent status, preoperative intra-aortic balloon pump (IABP), or cardiogenic shock (as defined by the European Society of Cardiology-Heart Failure guidelines [18]); (4) previous cardiac surgery.

The patients were divided into two groups (PAC and no-PAC) based on PAC insertion status. Only patients who had PAC inserted before or during surgery were assigned to the PAC group, while all other patients were assigned to the no-PAC group. The preoperative use of PAC was not random but at the discretion of the anesthesiologist. All anesthesiologists involved in the present study were experienced cardiac anesthesiologists. For OPCAB surgical patients, the anesthesiologist decides on PAC use or not depending on various factors, considering each potential combination of patients, surgical procedures, and practice settings in low-, moderate-, and high-risk categories [19]. For the patient aspect, the anesthesiologist’s main concern is the left ventricular function (left ventricular eject fraction, left ventricular end-diastolic diameter, left ventricular aneurysm), myocardial infarction (MI) within a month, severe pulmonary artery hypertension (PAH), if IABP was required before surgery, etc. With these factors, anesthesiologists will combine their experience to guide the application of PAC.

### 2.2 Data Collection

Data for the present study was obtained from the Hospital Information System and the Anesthesia Information Management System, which included detailed preoperative, intraoperative, and postoperative data on all hospitalized patients who underwent primary and isolated OPCAB surgery. Baseline characteristics (e.g., age, gender, height, weight, comorbidities, medications, serum creatine, and left ventricular ejection fraction), intraoperative data (e.g., the operative time, inotropic and vasoactive medication administrations, fluid infusion), and postoperative outcomes (e.g., death, complications, transfusion, mechanical ventilation duration (MVD), LOS in intensive care unit (ICU) and hospital, chest drainage duration, inotropic and vasoactive medication administrations, re-admission to ICU, reoperation, hospitalization costs) were extracted.

### 2.3 Primary and Secondary Outcomes

The primary outcome was the composite incidence of hospitalized death and complications (e.g., cardiac arrest, new-onset atrial fibrillation, pacemaker, myocardial infarction, low cardiac output, use of IABP and extracorporeal membrane oxygenation (ECMO), stroke, any other neurological events, renal replacement therapy, and pulmonary infection), which were widely used for evaluating the quality of CABG procedures [20, 21, 22]. The composite incidence of hospitalized complications was expressed as the number of adverse events during the index hospitalization. The analysis was at the individual patient level, with each type of complication counted only once [23]. Non-fatal myocardial infarction was classed as those newly occurring postoperatively. It was defined as any of the following in the medical record or electrocardiograph-documented Q waves that were 0.03 seconds in width and one-third or greater of the total QRS complex in 2 or more continuous leads [20]. Low cardiac output was defined as a cardiac index of 2.2 L/min/$m^{2}$ or mixed venous oxygen saturation <50% with the need for positive inotropic support. Stroke was defined as brain, spinal cord, or retinal cell death attributable to focal arterial ischemia [24]. Other neurological events were central neurological deficits that lasted more than 72 hours [25]. Renal replacement therapy was defined as requiring dialysis to treat chronic oliguria or anuria [26]. Pulmonary infection diagnosis requires a physician or senior practitioner documentation in the medical record according to laboratory findings (e.g., positive sputum culture results from transtracheal fluid of bronchial washings) and radiologic evidence (e.g., chest radiograph showing pulmonary infiltrates consistent with the diagnosis of pneumonia).

The secondary outcomes were postoperative recovery parameters (MVD, LOS in ICU and hospital, re-admission to ICU, and reoperation) and total hospitalization costs. Other comparison outcomes were intraoperative and postoperative fluid infusion, vasoactive drug, chest drainage duration, and blood product transfusion (red blood cells, plasma, platelets) between the two groups.

Blood products were transfused based on the hospital transfusion protocol. For example, the transfusion trigger for red blood cells (RBC) was hemoglobin (HB) <8.0 g/L. The indication for fresh frozen plasma (FFP) transfusion was diffuse bleeding with a 1.5-fold prolongation of prothrombin time from baseline values. The threshold for platelet concentrate (PC) transfusion was a platelet (PLT) count <50 × ${\mathrm{10}}^{9}$/L or PLT dysfunction. Reoperation when postoperative drainage volume was 300 mL/h for the first 2 hours or 200 mL/h for 4 consecutive hours. Removed the chest drainage tube when the drainage volume was <20 mL for 5~6 hours. Extubation criteria included: ① the patient was conscious, ② the gag, swallowing, and cough reflexes were fully restored, ③ the tidal volume and minute ventilation were normal, ④ no factors causing airway obstruction. The criteria for transfer from the ICU were that the tracheal tube had been removed, the patient was fully awake, could cough as instructed by the physician, and could do the conscious movement of the limbs. It was important to note that the ICU did not transfer out patients on weekends.

### 2.4 Statistical Analysis

Patient characteristics before and after matching were compared using *p* values; *p* < 0.05 between the two groups after propensity matching was considered statistically significant. If continuous variables were normally distributed, they were expressed as mean ± standard deviation and compared by student *t*-test/paired *t*-test. If the data were not normally distributed, they were described as median and quartile range and compared with paired Wilcoxon-Mann-Whitney test. D’Agostino’s skewness/kurtosis test was used to check whether the distribution characteristics of the variables conform to normality. Dichotomous variables were expressed in frequency and percentage and compared using the chi-square or Fisher exact test. Missing data were considered to be missing completely at random. The data of the two groups was also compared and analyzed using IBM SPSS version 26.0 software (SPSS Inc., Chicago, IL, USA).

## 3. Results

### 3.1 Baseline Characteristics

**Fig. 1.**
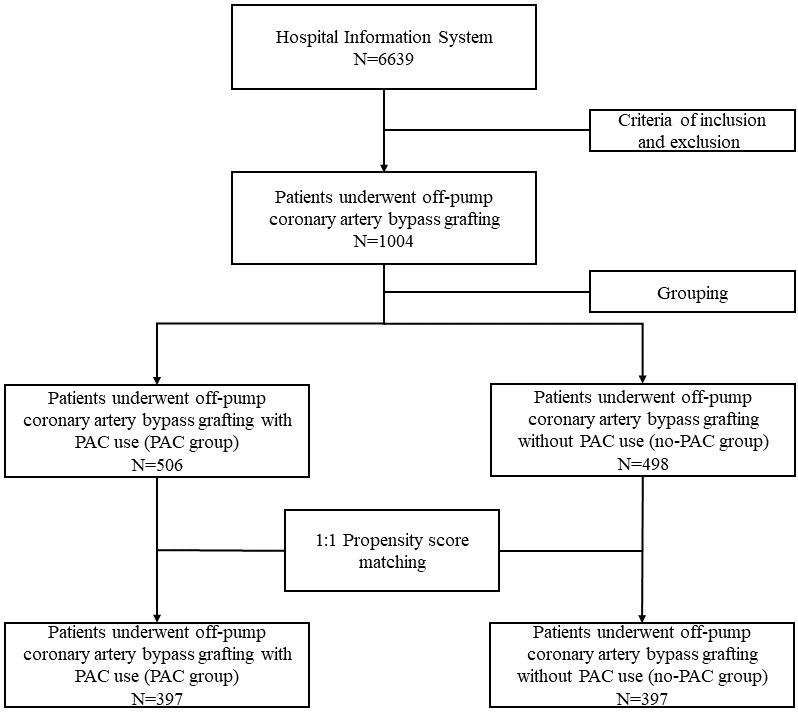
**Patient enrollment**. PAC, pulmonary artery catheter.

The patient enrollment is shown in Fig. 1. 1004 patients who underwent OPCAB surgery were identified, 700 patients (69.7%) had a three-vessel disease, 196 patients (19.5%) had left main trunk disease, and 506 patients (50.4%) had PAC use. Table 1 lists the demographic and clinical variables for the entire unmatched and matched cohorts, analyzed by PAC insertion status. In the entire cohort, the 1004 eligible patients showed no significant differences between the two groups concerning age, smoking, drinking, diabetes mellitus, hypertension, and other comorbidities. However, the patients in the PAC group had higher BMI (25.2 ± 3.2 vs. 26.5 ± 3.2, *p* < 0.001). There was no significant difference in baseline characteristics between PAC and no-PAC groups in the propensity-matching cohort.

**Table 1.** **Patient characteristics**.

		Entire cohort			Matched cohort		
		no-PAC (n = 498)	PAC (n = 506)	*p*	no-PAC (n = 397)	PAC (n = 397)	*p*
Demographic							
	Age, median (IQR)	63.0 (56.0, 68.0)	63.0 (55.0, 68.0)	0.632	62.00 (55.0, 68.0)	62.00 (55.0, 68.0)	0.890
	Female, n (%)	119 (23.9)	107 (21.1)	0.333	315 (79.3)	310 (78.1)	0.729
Risk factors							
	BMI, mean (SD)*	25.2 (3.2)	26.5 (3.2)	<0.001	25.8 (2.9)	25.8 (2.9)	0.993
	Smoking, n (%)	251 (50.4)	255 (50.4)	1.000	212 (53.4)	206 (51.9)	0.722
	Drinking, n (%)	242 (48.6)	235 (46.4)	0.536	203 (51.1)	195 (49.1)	0.619
	Hypertension, n (%)	330 (66.3)	345 (68.2)	0.562	268 (67.5)	261 (65.7)	0.652
	Hyperlipidemia, n (%)	420 (84.3)	415 (82.0)	0.369	330 (83.1)	323 (81.4)	0.577
	Diabetes, n (%)	201 (40.4)	198 (39.1)	0.738	153 (38.5)	161 (40.6)	0.611
	Pulmonary hypertension	4 (0.8)	9 (1.8)	0.277	3 (0.8)	4 (1.0)	1.000
Comorbidities							
	COPD, n (%)	5 (1.0)	6 (1.2)	1.000	4 (1.0)	5 (1.3)	1.000
	Atrial fibrillation, n (%)	16 (3.2)	17 (3.4)	1.000	13 (3.3)	10 (2.5)	0.672
	Myocardial infarction, n (%)	186 (37.3)	183 (36.2)	0.746	153 (38.5)	150 (37.8)	0.884
	PCI, n (%)	49 (9.8)	35 (6.9)	0.119	28 (7.1)	30 (7.6)	0.892
	Peripheral vascular diseases, n (%)	80 (16.1)	74 (14.6)	0.585	65 (16.4)	64 (16.1)	1.000
	Cerebral events, n (%)	45 (9.0)	35 (6.9)	0.261	31 (7.8)	33 (8.3)	0.896
Clinical profiles							
	LVEF, median (IQR)	60.0 (55.0, 64.0)	60.0 (55.0, 64.0)	0.791	60.0 (55.0, 64.0)	60.0 (55.0, 64.0)	0.977
	Serum creatine, median (IQR)	84.9 (75.0, 96.0)	84.1 (74.7, 97.0)	0.584	86.1 (77.0, 98.3)	84.1 (74.0, 97.7)	0.186
Medications							
	Aspirin, n (%)	85 (17.1)	102 (20.2)	0.239	72 (18.1)	82 (20.7)	0.419
	Clopidogrel, n (%)	92 (18.5)	94 (18.6)	1.000	78 (19.6)	81 (20.4)	0.859
	β-blockers, n (%)	279 (56.0)	294 (58.1)	0.547	224 (56.4)	226 (56.9)	0.943
	Calcium channel blocker, n (%)	259 (52.0)	272 (53.8)	0.623	211 (53.1)	201 (50.6)	0.523
	ACEI/ARB, n (%)	52 (10.4)	51 (10.1)	0.932	41 (10.3)	45 (11.3)	0.732
	Nitrates, n (%)	330 (66.3)	345 (68.2)	0.562	268 (67.5)	261 (65.7)	0.652
	Anti-diabetics, n (%)	201 (40.4)	198 (39.1)	0.738	153 (38.5)	161 (40.6)	0.611
	Statins, n (%)	420 (84.3)	415 (82.0)	0.369	330 (83.1)	323 (81.4)	0.577

ACEI, angiotensin-converting enzyme inhibitors; ARB, angiotensin receptor blockers; BMI, body mass index; *BMI = weight (kg)/${\mathrm{(height [m])}}^{2}$; COPD, chronic obstructive pulmonary disease; LVEF, left ventricular ejection fraction; PCI, percutaneous coronary interventions; IQR, interquartile range; PAC, pulmonary artery catheter.

### 3.2 Intraoperative Data

Table 2 presents intraoperative data for the entire and matched cohorts. The PAC group had a more intraoperative crystalloid volume and total fluid infusion volume (*p* < 0.001) than the no-PAC group for the entire cohort. However, patients in the matched PAC group were more likely to use epinephrine (8.3% vs. 12.8%, *p *= 0.05). Notably, the two groups had no significant difference regarding operative time and anesthesia time before and after matching.

**Table 2.** **Intraoperative data**.

		Entire cohort			Matched cohort		
		no-PAC (n = 498)	PAC (n = 506)	*p*	no-PAC (n = 397)	PAC (n = 397)	*p*
Operative time, median (IQR)		204.5 (176.0, 235.8)	206.0 (178.0, 238.0)	0.546	204.0 (175.0, 235.0)	202.0 (176.0, 235.0)	0.799
Anesthesia time, median (IQR)		253.5 (217.0, 286.0)	253.0 (220.0, 290.0)	0.585	255.0 (220.0, 285.0)	247.0 (218.0, 280.0)	0.275
Intraoperative fluid infusion							
	Crystalloid volume, median (IQR)	1000.0 (575.0, 1400.0)	1000.0 (700.0, 1500.0)	< ${\mathrm{0.001}}^{*}$	1000.0 (500.0, 1400.0)	1000.0 (600.0, 1500.0)	0.066
	Colloidal volume, median (IQR)	500.0 (100.0, 500.0)	500.0 (300.0, 500.0)	0.061	500.0 (100.0, 500.0)	500.0 (200.0, 500.0)	0.317
	Total infusion volume, median (IQR)	1500.0 (1000.0, 1825.0)	1500.0 (1025.0, 2000.0)	< ${\mathrm{0.001}}^{*}$	1500.0 (1000.0, 1900.0)	1500.0 (1000.0, 2000.0)	0.066
Inotropic/vasoactive agents							
	Epinephrine, n (%)	44 (8.8)	64 (12.6)	0.065	33 (8.3)	51 (12.8)	0.050
	Milrinone, n (%)	38 (7.6)	35 (6.9)	0.754	29 (7.3)	28 (7.1)	1.000
	Dopamine, n (%)	253 (50.8)	271 (53.6)	0.418	199 (50.1)	214 (53.9)	0.320
	Norepinephrine, n (%)	94 (18.9)	90 (17.8)	0.716	73 (18.4)	70 (17.6)	0.853
	Nitroglycerin, n (%)	328 (65.9)	335 (66.2)	0.962	276 (69.5)	262 (66.0)	0.324

IQR, interquartile range; PAC, pulmonary artery catheter. *indicates that *p* is statistically significant.

### 3.3 Postoperative Data

Table 3 presents postoperative data for entire and matched cohorts. In the entire cohort, the composite rate of mortality and mortalities occurred in 21.7% of the PAC group and 16.3 in the no-PAC group (*p* = 0.003). In the matched cohort, the composite rate of mortality and mortalities occurred in 22.4% of the PAC group and 16.9 in the no-PAC group (*p* = 0.061). Among the outcomes included in the primary composite endpoint, there was no difference in most observations between the two groups for the entire and matched cohort. Only the composite morbidity (*p* = 0.04) and new-onset atrial fibrillation (*p* = 0.008) were significantly different in the PAC group of the entire cohort but had no significant difference in the matched cohort.

**Table 3.** **Postoperative data**.

		Entire cohort			Matched cohort		
		no-PAC (n = 498)	PAC (n = 506)	*p*	no-PAC (n = 397)	PAC (n = 397)	*p*
Mortality and morbidities, n (%)		81 (16.3)	110 (21.7)	${\mathrm{0.033}}^{*}$	67 (16.9)	89 (22.4)	0.061
Mortality, n (%)		0 (0.0)	1 (0.2)	1.000	0 (0.0)	1 (0.3)	1.000
Any morbidity, n (%)		81 (16.3)	109 (21.5)	${\mathrm{0.040}}^{*}$	67 (16.9)	88 (22.2)	0.073
	New-onset atrial fibrillation, n (%)	0 (0.0)	9 (1.8)	${\mathrm{0.008}}^{*}$	0 (0.0)	5 (1.3)	0.073
	Cardiac arrest, n (%)	1 (0.2)	2 (0.4)	1.000	1 (0.3)	2 (0.5)	1.000
	Myocardial infarction, n (%)	29 (5.8)	35 (6.9)	0.562	22 (5.5)	28 (7.1)	0.465
	Low cardiac output, n (%)	7 (1.4)	7 (1.4)	1.000	6 (1.5)	6 (1.5)	1.000
	Pulmonary infection, n (%)	61 (12.2)	67 (13.2)	0.706	48 (12.1)	56 (14.1)	0.462
	Stroke, n (%)	9 (1.8)	15 (3.0)	0.320	8 (2.0)	13 (3.3)	0.376
	Any neurological events, n (%)	14 (2.8)	17 (3.4)	0.749	10 (2.5)	14 (3.5)	0.534
	Renal replacement therapy, n (%)	11 (2.2)	17 (3.4)	0.360	11 (2.8)	15 (3.8)	0.550
Pacemaker, n (%)		4 (0.8)	3 (0.6)	0.983	4 (1.0)	3 (0.8)	1.000
IABP, n (%)		1 (0.2)	4 (0.8)	0.379	1 (0.3)	3 (0.8)	0.616
ECMO, n (%)		7 (1.4)	9 (1.8)	0.826	6 (1.5)	9 (2.3)	0.602
MVD (h), median (IQR)		16.00 (13.00, 18.00)	15.00 (13.00, 18.00)	0.257	16.00 (13.00, 17.00)	15.00 (13.00, 18.00)	0.541
LOS in ICU (h), median (IQR)		61.00 (26.38, 93.25)	63.00 (28.00, 93.50)	0.634	62.00 (26.75, 93.00)	51.25 (23.00, 93.00)	0.805
LOS in hospital (d), median (IQR)		7.00 (7.00, 9.00)	7.00 (7.00, 9.00)	0.345	7.00 (7.00, 9.00)	7.00 (7.00, 9.00)	0.500
Hospitalization costs (RMB), median (IQR)		111823.48 (102965.84, 124892.44)	118884.51 (107370.11, 133418.28)	< ${\mathrm{0.001}}^{*}$	112071.52 (102192.40, 125116.20)	119559.34 (107504.62, 133432.17)	<0.001
Re-admission to ICU, n (%)		6 (1.2)	9 (1.8)	0.625	5 (1.3)	7 (1.8)	0.771
Reoperation, n (%)		4 (0.8)	7 (1.4)	0.562	2 (0.5)	6 (1.5)	0.286
Chest drainage time		4.00 (3.00, 5.00)	4.00 (3.00, 5.00)	0.383	4.00 (3.00, 5.00)	4.00 (3.00, 5.00)	0.426
RBC transfusion, n (%)		9 (1.8)	10 (2.0)	1	6 (1.5)	8 (2.0)	0.787
FFP transfusion, n (%)		11 (2.2)	13 (2.6)	0.867	11 (2.8)	9 (2.3)	0.821
Epinephrine, n (%)		44 (8.8)	64 (12.6)	0.065	33 (8.3)	51 (12.8)	0.050
Milrinone, n (%)		38 (7.6)	35 (6.9)	0.754	29 (7.3)	28 (7.1)	1.000
Dopamine, n (%)		253 (50.8)	271 (53.6)	0.418	199 (50.1)	214 (53.9)	0.320
Norepinephrine, n (%)		94 (18.9)	90 (17.8)	0.716	73 (18.4)	70 (17.6)	0.853
Nitroglycerin, n (%)		328 (65.9)	335 (66.2)	0.962	276 (69.5)	262 (66.0)	0.324

ECMO, extracorporeal membrane oxygenation; FFP, fresh frozen plasma; IABP, intra-aortic balloon pump; ICU, intensive care unit; IQR, interquartile range; LOS, length of stay; MVD, mechanical ventilation duration; RBC, red blood cell; RMB, ren min bi; PAC, pulmonary artery catheterization. *indicates that *p* is statistically significant. 1 RMB = 0.1401 USD.

In the entire and matched cohorts, there was no significant difference in the postoperative MVD, LOS in ICU and in hospital, the re-admission rate to ICU, reoperation, RBC transfusion, and FFP transfusion between the PAC and no-PAC groups. However, the hospitalization costs were higher (*p* < 0.001) in the PAC group. In the matched cohort, the PAC group had higher hospitalization costs (*p* < 0.001) and was more likely to use epinephrine (*p* = 0.05) than those without PAC insertion.

## 4. Discussion

Since its introduction in 1970 by Professor Jeremy Swan and William Ganz [1], PAC inserted at the bedside has been used in perioperative settings as a diagnostic tool and continuously monitors various physiological and hemodynamic parameters of critically ill surgical patients [3, 4, 5, 6, 7, 8, 9, 10, 11, 12, 13, 14, 15, 16, 17]. However, the effect of PAC-guided goal-directed treatment on morbidity and mortality has not been clearly proven, and PAC utilization has been shown to increase healthcare service and hospitalization costs [4, 9, 13, 14, 17]. The SUPPORT study involved medical and surgical patients and showed PAC utilization had increased mortality, LOS in ICU, and hospitalization costs [4]. This study raised benefit-risk concerns over PAC utilization and generated intense interest in social medicals and professional circles. The PAC-man study enrolled 1041 ICU patients from the United Kingdom (UK) and showed no difference in hospital mortality between patients managed with or without PAC [6]. Subsequent consensus statements recommended redoubled efforts at education regarding the use of pulmonary-artery catheters. Thus, PAC use has declined in ICU and non-cardiac surgery settings. For example, one study reported that PAC use decreased by 65% from 5.66 per 1000 medical admissions in 1993 to 1.99 per 1000 in 2004 in surgical and critically ill patients [27]. Another survey reported PAC utilization in heart failure (HF) patients decreased by 67.8% from 6.28 per 1000 admissions in 1999 to 2.02 per 1000 admissions in 2013 [28]. However, other studies indicated that PAC was still highly used in cardiovascular surgery. For example, Judge and colleagues surveyed 6000 members of the Society of Cardiovascular Anesthesiologists (SCA) and showed that most respondents preferred using PAC for most cardiac patients [8]. Similarly, Brovman and colleagues reported that PAC utilization increased from 2010 to 2014 in cardiac surgical patients [29].

The present study analyzed records of patients undergoing OPCAB surgery with or without PAC to estimate the impact of PAC use on short-term clinical outcomes and found PAC utilization was associated with a higher likelihood of using epinephrine during intra- and postoperative settings and higher hospitalization costs. Still, it did not affect hospitalization mortality, and no significant intergroup differences in numerous adverse outcomes were noted, including stroke, neurological events, and renal replacement therapy. The effect of PAC utilization on long-term prognosis has also been reported. Xu and colleagues [17] reported that PAC use in CABG patients was neither associated with perioperative mortality and major complications, nor with long-term mortality and major adverse cardiac and cerebrovascular events. Particularly for renal replacement therapy, acute kidney injury was a strong predictor of clinical outcome after cardiac surgery [30]. Besides, PAC utilization also has limited benefits for patients undergoing coronary or valvular surgery. Brovman and colleagues reported that PAC utilization was not associated with improved operative mortality in the entire cohort of (n = 11,820), matched cohort (n = 7038), and in the recent heart failure, mitral valve disease, or tricuspid insufficiency subgroups. LOS in the ICU was longer, and there were more packed RBC transfusions in the PAC group; however, postoperative outcomes were similar, including stroke, sepsis, and new renal failure [29]. Thus, PAC should only be used when it is indicated.

It is worth noting that PAC is a user-dependent diagnostic and surveillance technique and not a treatment per se. Patients who underwent OPCAB surgery may benefit from timely-targeted and effective interventions guided by the comprehensive and real-time parameters provided by PAC. The anesthesiologists’ proficiency and experience with PAC were essential. Professor Jeremy Swan [31], the inventor of PAC, suggested that physicians place at least 50 PACs annually to maintain proficiency. Schwann and colleagues reported that PAC utilization during CABG was associated with increased end-organ failure rate and hospitalization death, possibly due to overtreatment with positive inotropic drugs and intravenous fluids in the PAC cohort [16]. This study also showed increased fluid infusion and epinephrine use in the PAC group. Tuman *et al*. [11] hypothesized that the PAC group was more likely to use drugs, partly reflecting how monitoring and unnecessary information influenced treatment without significantly altering outcomes. These results highlighted the risk of overtreating abnormal physiological parameters.

In 2003, the American Society of Anesthesiologists (ASA) updated the practice guidelines for PAC utilization, advising that the appropriate use of PAC should be based on three aspects, namely patient factors, surgery factors, and practice factors [19]. In 2021, the Chinese Society of Anesthesiology (CSA) recommended the indications of PAC utilization for cardiac surgical patients, including left ventricular systolic dysfunction (eject fraction <30%), right ventricular systolic dysfunction, left ventricular diastolic dysfunction, acute ventricular septal perforation and left ventricular assist device. In the present study, the administration of PAC was based on the anesthesiologist’s assessment. The anesthesiologist decides on PAC utilization or not, depending on various factors. Left ventricular function was the most commonly considered factor. Left ventricular dysfunction was associated with a high incidence of death and complications after cardiac surgery due to hemodynamic instability and potentially nonviable myocardium [32]. The key question was not whether PAC utilization improves outcomes but whether the treatment taken by the anesthesiologist based on PAC data improves outcomes. If a trained physician considers invasive hemodynamic data to be necessary to treat a particular patient, a PAC is justified.

Although the ideal evaluation of PAC in clinical practice is a randomized controlled trial (RCT), this effort is time-consuming, expensive, and has limited generalization [33].

We found that during the retrieval process, there was only one RCT study on PAC in CABG surgery, and it was published in 1989, with a small sample size (226) and selection bias [34]. In fact, most were observational cohort studies. This is the first and the largest survey to evaluate the relationships between PAC in OPCAB surgery and clinical outcomes in China. The results showed no differences in hospitalization outcomes between the OPCAB patients who used PAC and those who did not, except for significantly greater intra- and post-operative epinephrine use among the PAC group.

## 5. Conclusions

The current study demonstrated no apparent benefit or harm for PAC use during OPCAB surgery. However, PAC utilization in OPCAB surgery was more expensive.

## 6. Limitations

The limitations of this study should be acknowledged. First, it is a retrospective study performed at a single center and using existing databases. The cohort could have been affected by selection bias. However, propensity score matching is used to overcome this discrepancy in the hopes of making each group more comparable regarding baseline risk. Fortunately, there are no significant differences in baseline characteristics between the PAC and no-PAC groups before propensity score-matching. Second, surgeons, anesthesiologists, and critical care physicians may have different levels of familiarity and expertise with PAC utilization, which may introduce some heterogeneity in the perioperative management of this population. Currently, there is no institutional protocol for managing hemodynamic and physiological parameters measured by PAC, which may lead to heterogeneity in perioperative care in this population. Furthermore, the effective use of PAC is an elusive variable that cannot be captured in this study. Moreover, as a single institution’s experience, all the subjects enrolled in the trial were from the same hospital in the same region, and the implications of these findings may not be fully generalizable.

## Data Availability

The article’s data will be shared on reasonable request with the corresponding author.
